# Short-chain fatty acids regulate systemic bone mass and protect from pathological bone loss

**DOI:** 10.1038/s41467-017-02490-4

**Published:** 2018-01-04

**Authors:** Sébastien Lucas, Yasunori Omata, Jörg Hofmann, Martin Böttcher, Aida Iljazovic, Kerstin Sarter, Olivia Albrecht, Oscar Schulz, Brenda Krishnacoumar, Gerhard Krönke, Martin Herrmann, Dimitrios Mougiakakos, Till Strowig, Georg Schett, Mario M. Zaiss

**Affiliations:** 1 Department of Internal Medicine 3, Rheumatology and Immunology, Friedrich-Alexander-University Erlangen-Nürnberg (FAU) and Universitätsklinikum Erlangen, 91054 Erlangen, Germany; 2 Division of Biochemistry, Department of Biology, Friedrich-Alexander University Erlangen-Nürnberg (FAU), 91054 Erlangen, Germany; 3 Department of Internal Medicine 5, Hematology and Oncology, Translational Research Center, University Hospital Erlangen, 91054 Erlangen, Germany; 4 Helmholtz Centre for Infection Research, 38124 Braunschweig, Germany

## Abstract

Microbial metabolites are known to modulate immune responses of the host. The main metabolites derived from microbial fermentation of dietary fibers in the intestine, short-chain fatty acids (SCFA), affect local and systemic immune functions. Here we show that SCFA are regulators of osteoclast metabolism and bone mass in vivo. Treatment of mice with SCFA as well as feeding with a high-fiber diet significantly increases bone mass and prevents postmenopausal and inflammation-induced bone loss. The protective effects of SCFA on bone mass are associated with inhibition of osteoclast differentiation and bone resorption in vitro and in vivo, while bone formation is not affected. Mechanistically, propionate (C3) and butyrate (C4) induce metabolic reprogramming of osteoclasts resulting in enhanced glycolysis at the expense of oxidative phosphorylation, thereby downregulating essential osteoclast genes such as TRAF6 and NFATc1. In summary, these data identify SCFA as potent regulators of osteoclast metabolism and bone homeostasis.

## Abstract (web summary)

Short-chain fatty acids (SCFA) are a main class of metabolites derived from fermentation of dietary fibre in the intestine. Here, the authors show that dietary administration of SCFA is associated with inhibition of osteoclast differentiation, increased bone mass, and reduced pathological bone loss in mice.

## Introduction

Several reports highlighted the immunomodulatory capacities of short-chain fatty acid (SCFA)^1^. As immune activation is intimately linked to bone homeostasis^2–4^, we hypothesized that SCFA may influence bone homeostasis thereby providing a direct mechanistic link between the gut microbiota and bone. In support of the clinical relevance of this concept, recent studies suggested that presence or absence of specific bacterial species influence the course of inflammatory bone diseases, including rheumatoid arthritis (RA)^5–7^ and ankylosing spondylitis^8^. Furthermore, it was shown that fiber-rich diet, the main fermentable source for SCFA, can ameliorate bone destruction in RA^9^. These observations prompted us to investigate the role of SCFA on bone during steady-state conditions as well as in the case of pathological bone loss during menopause and inflammation. Studies addressing the role of the gut microbiota on bone homeostasis by using germ-free (GF), antibiotic-treated or mono-colonized mice reported contradicting findings^10–15^. These discrepancies may be explained by different microbial exposure of the mice used for the experiments. Therefore, we tested whether the secreted metabolites of the gut microbiota rather than the microbiota per se may explain these effects. To test this hypothesis, we used three independent experimental approaches (direct supplementation of SCFA, high-fiber diet (HFD), and bacterial transfer) and found consistent beneficial effects on steady state and postmenopausal bone metabolism highly related to SCFA levels. It should be also mentioned that the source and biological function of SCFA is essentially different from long-chain fatty acids, which influence insulin metabolism and where effects on bone have been suggested^16^.

Here we show that the SCFA C3 and C4 have a beneficial impact on bone homeostasis and alleviated disease in two models of inflammatory arthritis. At the molecular level, the direct impact of SCFA on bone homeostasis is dependent on early metabolic reprogramming of pre-osteoclasts. Moreover, immunosuppressive mechanisms induced by SCFA also contribute to the attenuation of inflammation by inducing regulatory T cells (Tregs). Hence, our findings indicate a crucial role of SCFA in bone metabolism and immune responses attenuating the severity of arthritis.

## Results

### SCFA and HFD increase systemic bone density

**Fig. 1:**
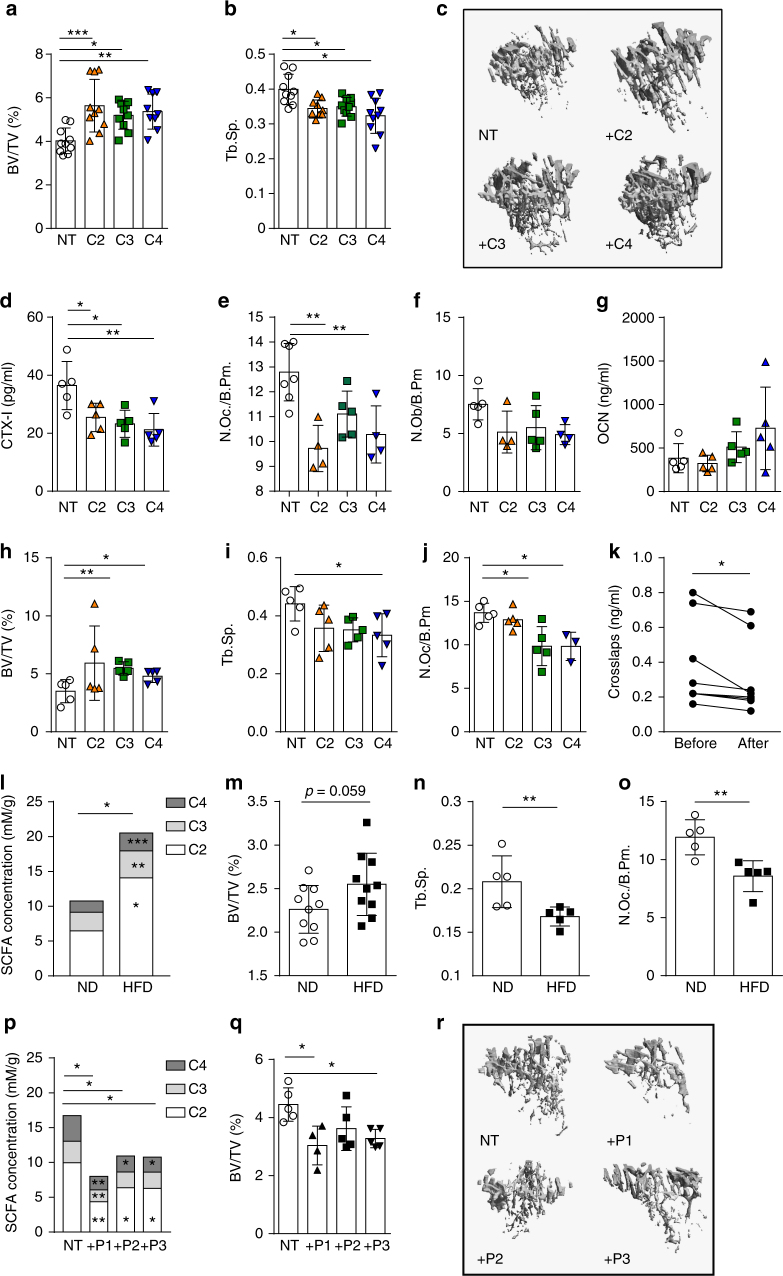
SCFA improve systemic bone mass under steady-state conditions. WT mice not treated (NT) or treated with C2 (acetate), C3 (propionate), or C4 (butyrate) in the drinking water for 8 weeks were analyzed by µCT for **a** bone volume per total volume (BV/TV) and **b** trabecular separation (Tb.Sp.). **c** Representative µCT images of the trabecular part of tibial bone of mice not treated (NT) or treated with SCFA (C2/C3/C4). **d** Serum CTX-I levels as marker for bone resorption measured by ELISA. **e** Immunohistological analysis of TRAP-stained osteoclasts in the tibia of SCFA-exposed WT mice showing the number of osteoclasts per analyzed bone parameter (N.Oc./B.pm). **f** Immunohistological analysis of osteoblasts in the tibia of SCFA-exposed WT mice showing N.Ob./B.pm. **g** Serum osteocalcin (OCN) bone formation marker measured by ELISA. **h-i**. RAG1^−/^^−^ mice NT or treated with C2/C3/C4 in the drinking water for 8 weeks were analyzed by µCT for **h** BV/TV and **i** Tb.Sp. **j**. Immunohistological analysis of TRAP-stained osteoclasts in the tibia of SCFA-exposed RAG1^−/^^−^ mice showing N.Oc./B.pm. **k** Serum Crosslaps bone resorption marker measured by ELISA before and after 2 weeks of sodium propionate treatment in healthy control human subjects. **l** Gas-chromatographic mass-spectrometric analysis of SCFA concentrations in cecal samples expressed as mM per gram feces. **m**,** n** WT mice fed with special high-fiber diet (HFD) and normal control diet (ND) analyzed by µCT for **m** BV/TV and **n** Tb.Sp. **o** Immunohistological analysis of TRAP-stained osteoclasts in the tibia of WT mice fed with special HFD and ND showing N.Oc./B.pm. **p**–**r** WT mice receiving adoptive transfer of isolated *Prevotella* strains analyzed by **p** gas-chromatographic mass-spectrometric analysis for SCFA concentration in cecal samples expressed as mM per gram feces and **q** by µCT for BV/TV. **r** Representative µCT images of the trabecular part of tibial bone of NT mice or receiving adaptive transfer of isolated *Prevotella* (P1/P2/P3). Pictures are representative of at least two independent experiments. Data are expressed as the mean ± s.d. Statistical difference was determined by one-way ANOVA or Student's *t*-test. *p < 0.5; **p < 0.01; ***p < 0.001

To determine whether SCFA affect bone metabolism, we analyzed bone mass in C57BL/6J (wild-type (WT)) mice either fed with or without C2/C3/C4 supplementation in the drinking water for 8 weeks. After 8 weeks, serum SCFA concentrations in treated mice were significantly increased (Supplementary Fig. 1a). Micro-computed tomography (µCT) analysis of tibial bone showed that SCFA treatment significantly increased bone mass as shown by increased bone volume per tissue volume (BV/TV) and decreased trabecular separation (Tb.Sp) (Fig. 1a–c). CTX-I serum level, a marker for bone resorption, was significantly decreased (Fig. 1d), and SCFA treatment led to a significant decrease of bone-degrading osteoclasts as shown by histomorphometric analysis (Fig. 1e). In contrast, markers for bone formation such as osteoblast numbers, serum osteocalcin (OCN) levels (total and decarboxylated) and mineral apposition rate and bone formation rate per bone surface remained unchanged (Fig. 1f, g and Supplementary Fig. 1b, c).

As SCFA were shown to act on immune cells, especially to induce Tregs^17–19^, we analyzed Treg numbers in the spleens and found increased numbers after SCFA treatment while serum cytokine levels remained largely unchanged, except for C4 treatment that impacted on interleukin (IL)-10, interferon-γ (IFNγ), transforming grotwh factor-β (TGFβ) as well as on tumor necrosis factor-α (TNFα) levels (Supplementary Fig. 1d, e). Increased Tregs may explain the bone effects of SCFA since Treg were shown to suppress osteoclasts and increased bone mass^20–22^. To exclude this possibility, we treated RAG1^−^^/−^ mice with SCFAs and also observed an increase in BV/TV along with decreased trabecular separation (Tb.Sp.) and osteoclast numbers (Fig. 1h–j). Interestingly, osteoclast numbers were not decreased in RAG1^−/−^ mice after C2 treatment, pointing toward a different suppressive mechanism. Although we cannot exclude other possible indirect mechanisms on innate immune cells, we can clearly exclude the impact of T and B cells with these experiments. Also, a 14-day nutritional supplementation of sodium propionate (Propicum) in healthy humans significantly decreased serum parameters for bone resorption (Fig. 1k) while leaving OCN levels unchanged (Supplementary Fig. 1f).

Next, we investigated whether diets rich in fermentable, indigestible fibers, which increase SCFA levels in the cecum and serum (Fig. 1l and Supplementary Fig. 1g), exert similar effects on bone. In accordance with direct SCFA supplementation, an 8-week exposure to fiber-rich diet (Supplementary Table 1) led to an increased bone mass (Fig. 1m) and decreased trabecular separation (Fig. 1n) associated with decreased osteoclast numbers and CTX-I serum levels (Fig. 1o and Supplementary Fig. 1h). At the same time, OCN serum levels and osteoblast numbers remained unchanged (Supplementary Fig. 1i, j). Although Treg numbers were also increased after HFD treatment, serum cytokine levels did not show significant changes (Supplementary Fig. 1k, l). Of note, no differences in weight gain were observed in the feeding experiments, either with SCFA or HFD (Supplementary Fig. 1m).

We next addressed whether the transfer of specific bacterial species and respective changes of gut microbiota and its secreted metabolites could also impact bone mass. Enhanced abundance of *Prevotella copri* has been described in early RA^7^. We therefore transferred three representative species from the genus *Prevotella* separately into WT mice and analyzed systemic bone mass 8 weeks later. Transfer of all *Prevotella* spp. significantly increased osteoclast numbers (Supplementary Fig. 1n) and the relative abundance of *Prevotella* spp. durably for 8 weeks (Supplementary Fig. 1o, p) but decreased SCFA levels (Fig. 1p) and systemic bone mass in the recipient mice (Fig. 1q, r), thus highlighting the impact of disease-relevant changes in the microbiome and subsequently in SCFA levels on bone homeostasis. This change in bone mass after bacterial transfer seems to be specific for *Prevotella* spp. as transfer of representative bacteria from taxonomically related families *Bacteroidaceae* and *Muribaculaceae* did not show similar effects (Supplementary Fig. 1q).

### SCFA change the metabolic state of pre-osteoclasts

**Fig. 2:**
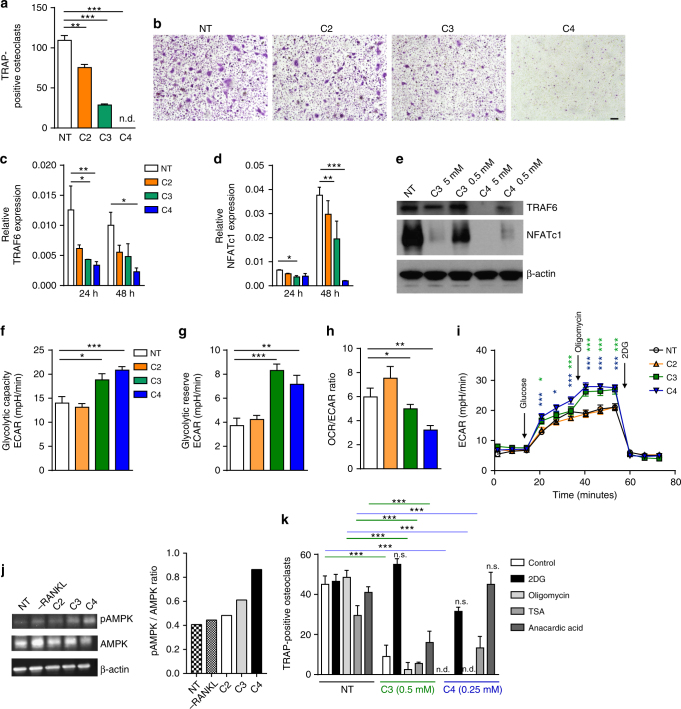
SCFA suppress osteoclastogenesis by changing cellular metabolism. **a** Quantification and **b** representative microphotographs of multinucleated TRAP-positive osteoclasts from in vitro bone marrow cell cultures stimulated with M-CSF and RANKL and treated with 0.5 mM C2, C3, C4, or left untreated (not treated, NT). Scale bar, 1000 µm. **c**–**d** Real-time PCR analysis of osteoclast marker genes **c** TRAF6 and **d** NFATc1 during osteoclast differentiation after 24 and 48 h of SCFA exposure. **e** Western blot analysis of TRAF6 and NFATc1 expression after C3 and C4 stimulation for 48 h. **f**, **g** Analysis of the extracellular acidification rate (ECAR) by glycolysis measured with a Seahorse real-time metabolic analyzer: **f** glycolytic capacity and **g** glycolytic reserve. **h** Ratio of ECAR to oxygen consumption rate (OCR), the latter resembling mitochondrial activity. **i** Time course of ECAR values in osteoclasts expose to SCFA. **j** Representative western blots of total AMPK and phosphorylated AMPK (pAMPK) in osteoclasts not treated (NT) or treated with SCFA (C2/C3/C4) (left panel) and quantification of the pAMPK/AMPK ratio (right panel). **k** Quantification of multinucleated TRAP-positive osteoclasts from in vitro bone marrow cell cultures stimulated with M-CSF and RANKL and treated with 0.5 mM C3 or 0.25 mM C4 or left untreated (not treated, NT) and incubated with 0.1 mM 2-deoxy-D-glucose (2DG), 2.5 nM oligomycin, 5 nM trichostatin A (TSA), or 10 µM anacardic acid. Pictures are representative of at least three independent experiments. Data are expressed as the mean ± s.d. Statistical difference was determined by one-way or two-way ANOVA. *p < 0.5; **p < 0.01; ***p < 0.001

Our in vivo data indicated that SCFA modulate bone metabolism, resulting in significant decreased bone resorption. In vitro, osteoclast differentiation assays showed that concentrations from 0.03 mM of C2 and 0.015 mM of C3/C4 suppressed osteoclast differentiation (Fig. 2a, b), and this range of concentration was found in pooled bone marrow fluids from two individual experiments after SCFA feeding, namely, around 0.4 mM for C3 and 0.15 mM for C4 after adjusting measured concentrations in flushed bone marrow samples to what we experimentally found in the bone marrow fluid volume from repeated measurements of 5.5 ± 1.34 µl (*n* = 5) (Supplementary Fig. 2a, b). The suppressive capacity of C2/C3/C4 on osteoclast differentiation and bone resorption was independent of the free fatty acid surface receptors FFA3 (GPR41) and FFA2 (GPR43) (Supplementary Fig. 2c, d). Mechanistically, C3 and C4 significantly suppressed two essential osteoclastogenic signaling components, *TRAF6* and *NFATc1*, at early time points after receptor activator of nuclear factor-κB ligand (RANKL) stimulation (Fig. 2c–e). Previous in vitro studies using C4 as HDAC inhibitor for influencing bone cells showed inhibition of osteoclasts while results were inconsistent in osteoblasts^23–26^. While our in vivo data showed no consistent effects of SCFA on osteoblasts and bone formation, C3 and C4 strongly suppressed osteoclast differentiation.

Cell metabolism governs different cell functions^27^ and during osteoclast differentiation several sequential metabolic changes happen^20,28,29^. For example, differentiation from precursors to mature osteoclasts depends on oxidative phosphorylation, whereas bone resorption by mature osteoclasts depends on glycolysis. Here we show that C3/C4 stimulation significantly induced glycolysis in osteoclast precursors after 48 h but left oxidative phosphorylation unchanged (Fig. 2f–i and Supplementary Fig. 2e). In fact, increased glycolysis has already been associated with downregulation of TRAF6 in other cells^30^. Of note, these metabolic shifts were also observed in osteoclast assays of single GPR41, GPR43, and GRP41/43 double knockout (KO) mice, although SCFA receptors are expressed on osteoclasts (Supplementary Fig. 2f, h). Western blot analysis to detect energy stress after C3 and C4 treatment by investigating the activation of AMPK reveals a higher pAMPK/AMPK ratio (Fig. 2j). Moreover, specific blocking of glycolysis by 2-deoxy-D-glucose (2DG) only during the first critical 48 h during osteoclastogenesis in the presence of C3 or C4 prevented the suppressive effect of C3 and C4 (Fig. 2k). Addition of C3 and C4 at later time points had no significant effects on osteoclast differentiation (data not shown). Together, these results demonstrate that treatment of osteoclast precursors with C3 and C4 shifts their metabolism at early time points (24–48 h) during osteoclast differentiation toward glycolysis, causing cell stress and thereby preventing osteoclast differentiation.

### SCFA protect from postmenopausal bone loss

**Fig. 3:**
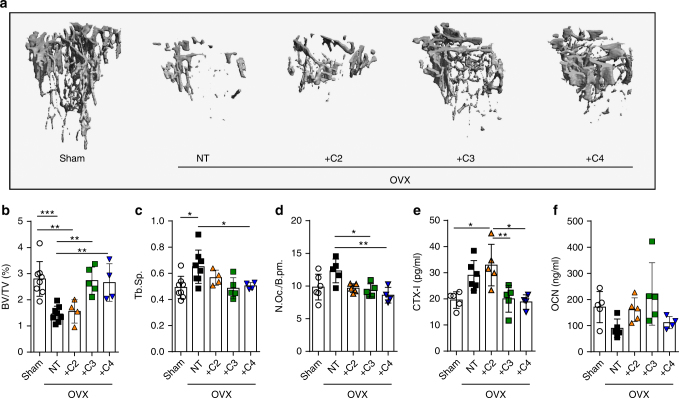
SCFA protect from postmenopausal bone loss. **a** Representative µCT images of the trabecular part of tibial bone of WT sham operated (Sham) or ovariectomized (OVX) mice fed with C2, C3, or C4 in the drinking water for 8 weeks or left untreated (not treated, NT). **b** Bone volume per total volume (BV/TV) and **c** trabecular separation (Tb.Sp.) analyzed by µCT. **d** Immunohistological analysis of TRAP-stained osteoclasts in the tibia of mice showing the number of osteoclasts per analyzed bone parameter (N.Oc./B.pm). **e** Serum CTX-I bone resorption and **f** osteocalcin (OCN) bone formation marker levels measured by ELISA. Data are expressed as the mean ± s.d. Pictures are representative of at two independent experiments. Statistical difference was determined by one-way ANOVA. *p < 0.5; **p < 0.01; ***p < 0.001

To test whether these findings could be exploited as novel strategy in the treatment of pathological bone loss, we investigated the impact of SCFA on postmenopausal bone loss induced after ovariectomy (OVX). Treatments with C3 and C4 effectively prevented OVX-induced bone loss (Fig. 3a–c). At the cellular level, we observed a complete rescue of OVX-induced osteoclast formation after C3 and C4 treatment (Fig. 3d). The OVX-induced increase in CTX-I concentrations in the serum was also completely blocked (Fig. 3e). In contrast, OCN levels remained unchanged (Fig. 3f and Supplementary Fig. 3a). Serum SCFA concentrations were significantly increased to the range observed for in vitro suppressive capacities of osteoclast differentiation (Supplementary Figs. 3b and 2a). Again, no significant changes in body weight or serum cytokine levels were detected (Supplementary Fig. 3c, d). Of note, no positive effects on BV/TV were observed after HFD feeding experiments during the OVX mouse model (Supplementary Fig. 3e).

### SCFA mitigate arthritis and protect from bone loss

**Fig. 4:**
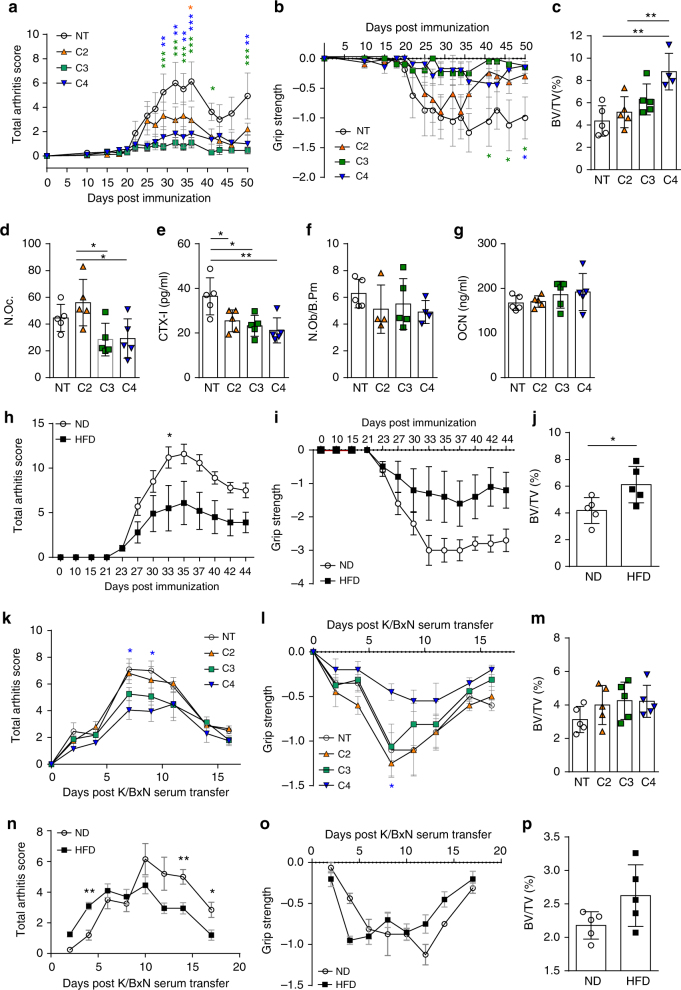
SCFA mitigate arthritis and protect from inflammatory bone loss. **a**–**j** Collagen-induced arthritis (CIA) model; **k**–**p** K/BxN serum-induced arthritis (SIA) model. **a** Joint swelling and **b** grip strength scores in mice induced for CIA and not treated (NT) or treated with C2 (acetate), C3 (propionate), or C4 (butyrate) in the drinking water. **c** µCT analysis of tibial bone mass showing bone volume per total volume (BV/TV). **d** Immunohistological analysis of TRAP-stained osteoclasts in the tibia of mice showing total osteoclast cell numbers per paw. **e** Serum CTX-I bone resorption marker level measured by ELISA. **f** Immunohistological analysis showing the number of osteoblasts per analyzed bone parameter in the tibia (N.Ob./B.pm). **g** Osteocalcin (OCN) bone formation marker level measured by ELISA. **h** Joint swelling and **i** grip strength scores in mice induced for CIA and fed with special high-fiber diet (HFD) or normal control diet (ND). **j** µCT analysis of tibial bone mass showing BV/TV. **k** Joint swelling and **l** grip strength scores in mice induced for SIA and not treated (NT) or treated with C2/C3/C4 in the drinking water. **m** µCT analysis of tibial bone mass showing BV/TV. **n** Joint swelling and **o** grip strength scores in SIA mice fed with special HFD or ND. **p** µCT analysis of tibial bone mass showing BV/TV. Data are expressed as the mean ± s.d. expect for **a**, **b**, **h**, **I**, **k**, **l**, **n**, and **o** where mean ± s.e.m. is shown. Pictures are representative of at least two independent experiments. Statistical difference was determined by one-way ANOVA, two-way ANOVA, or Student's *t*-test. *p < 0.5; **p < 0.01; ***p < 0.001

As early as 1979, it was shown that GF rats developed exacerbated arthritis^31^. Later reports revealed that not all types of bacteria have regulatory effects on arthritis. Pro-arthritogenic effects of certain bacterial strains were shown after mono-colonization of GF mice^32,33^. Furthermore, studies performed in gnotobiotic mice showed discrepant results with either antiarthritogenic^34^ or pro-arthritogenic^35^ effects of defined intestinal microbes. Therefore, we addressed the role of SCFA and HFD on inflammatory bone loss in two experimental arthritis models. C3/C4 treatment significantly attenuated the severity of inflammation in the collagen-induced arthritis (CIA) mouse model (Fig. 4a, b), and systemic bone mass was increased after C3/C4 treatment (Fig. 4c). Decreased osteoclast numbers in the affected paws and serum CTX-I levels were decreased, whereas osteoblast numbers and OCN serum levels remained unchanged (Fig. 4d, e). SCFA serum concentrations were significantly increased, whereas no effect on weight gain was observed (Supplementary Fig. 4a, b). Furthermore, osteoclast-specific gene expression in the bones was significantly downregulated after treatment with C3/C4 (Supplementary Fig. 4c). Of note, the observed effects seem to be largely independent of GPR43 as treatment with C3/C4 also maintained bone mass and suppressed osteoclast-specific genes in *GPR43* ^*−/−*^ mice (Supplementary Fig. 4d–h). Similar effects were observed in the CIA mouse model after HFD feeding (Fig. 4h–j) and in the K/BxN serum-induced mouse (SIA) model after SCFA supplementation in the drinking water (Fig. 4k–m), which significantly increased serum SCFA concentrations (Supplementary Fig. 4i). Furthermore, HFD feeding also protected from inflammatory bone loss during the SIA mouse model (Fig. 4n–p). In all performed experimental arthritis models, no change in weight gain was observed (Supplementary Fig. 4j, k).

## Discussion

In previous studies, we and others could show that microbiota, or their metabolites, regulate innate and adaptive immune responses^36–38^. Here we show a hitherto undiscovered role of SCFA on bone homeostasis. Our data suggest that microbial homeostasis in the gut associated with adequate production of SCFA is an important regulatory element in determining bone composition in mice. Hence, not necessarily the microbial species per se but rather the composition of secreted microbial metabolites, in particular SCFA, appear to link gut and bone homeostasis.

There are only few studies yet having addressed the role of the gut microbiota on bone homeostasis studying GF, antibiotic-treated or mono-colonized mice and reporting contradictory findings^10–15,39^. These discrepancies may be explained by different microbial reconstitution, exposure time, concentrations of nutritional supplements, and mice strains used for the experiments. Earlier reports discussed the possibility that SCFA might be responsible for the observed beneficial effects on bone after consumption of fiber-rich diets (inulin-type fructans^40^ and galactooligosaccharides^41^). Here we used three independent experimental approaches (direct supplementation of SCFA, HFD, and bacterial transfer) and found consistent beneficial effects on steady state, postmenopausal, and inflammatory bone metabolism highly correlated to increased SCFA levels.

Used concentrations in the in vitro experiments proved to be physiological as they were in the range of the increased SCFA concentrations in the serum and bone marrow samples after direct supplementation of SCFA or HFD feeding. Interestingly, steady-state SCFA concentrations in the bone marrow were higher than the lowest dilution used in our in vitro osteoclast suppression assays pointing toward a potential persistent suppressive capacity of SCFA during steady-state conditions. But this clearly needs to be further evaluated as our in vitro assays certainly neglect the complex situation found in the bone marrow niche with its multitude of factors impacting on the osteoclast differentiation. This is why we argue here that the balance between pro-osteoclastic and antiosteoclastic factors need to be taken into account. The mere steady-state concentrations are not that revealing, but what makes the difference is the increase over the steady-state concentrations.

In consequence, therapeutic supplementation of SCFA or diets increasing the endogenous production of SCFA may provide a powerful instrument to balance osteoclast activity and inhibit bone resorption. Even more importantly, in case of inflammatory bone loss the combination of anti-inflammatory immune-regulatory properties of SCFA together with the direct inhibition of osteoclast activity may be particularly useful. In this context, the benefits of the so-called Mediterranean-type diets, which are rich in fibers causing elevated levels of SCFA^42^ in managing human inflammatory arthritis, may be explained.

## Methods

### Mice and treatments

For all experiments, exept the OVX model, 8-week-old female mice were used unless stated otherwise. Five-to-six-week-old WT C57BL/6 and DBA/1J female mice (Charles River) were acclimated for 1 week, followed by a 2-week co-housing period before the experiments started. WT C57BL/6J used in the commensal transfer experiments were inbred WT C57BL/6N mice from the Helmholtz Centre for Infection Research, Braunschweig, Germany as indicated in the respective methods section. GPR41/43 double KO mice were kindly provided by Professor Stefan Offermanns (Max-Planck Institute, Bad Nauheim, Germany) and Dr. Tobias Junt (Novartis Pharma AG, Basel, Switzerland) provided the GPR41 and GPR43 single KO mice. All mice were maintained under specific pathogen-free conditions at the Präklinisches Experimentelles Tierzentrum (PETZ) Erlangen, Germany and approved by the local ethics authorities of the Regierung of Unterfranken (#55.2-2532-2-424). Supplementation of SCFA (all Sigma Aldrich, Germany) was done in the drinking water at a final concentration of 150 mM and changed every 3 days; control mice received pH and sodium-matched water. Specially adjusted fiber-rich diet (Supplementary Table 1) and control diet were purchased from Ssniff (Ssniff Spezialdiäten GmbH; Germany) and were provided ad libitum starting 2 weeks before the experiment and throughout the study.

### Bacteria growth and mice colonization

Three novel *Prevotella* species (*Prevotella* nov. P1, *Prevotella* nov. P2 and *Prevotella* nov. P3), *Bacteroides sartorii*, and *Muribaculum* sp. nov. have been isolated from the intestinal content of mice (data unpublished). All isolates were cultured anaerobically in Brain Heart Infusion broth, supplemented with 10% fetal bovine serum and 0.5 g/l vitamin K, for 48 h at 37 °C. WT C57BL/6N mice devoid of *Prevotella* spp., *Bacteroides sartorii*, or *Muribaculum* sp. nov. in their intestinal microbiota were bred and maintained in a specific-pathogen-free facility at Helmholtz Centre for Infection Research. For colonization experiments, 6–7-week-old female mice were colonized with one of each isolates via oral gavage. After 6–8 weeks, cecum content and serum samples were collected and stored at −80 °C until processing. Tibia and femurs were fixed in 4% formalin for 24 h and stored in 70% ethanol.

### DNA isolation and 16S rRNA microbial community analysis

Fresh stool samples of mice were collected and immediately stored at −20 °C. For DNA-based 16S rRNA gene sequencing, DNA was extracted according to established protocols using a method combining mechanical disruption (bead-beating) and phenol/chloroform-based purification^43^. Briefly, sample was suspended in a solution containing 500 µl of extraction buffer (200 mM Tris, 20 mM EDTA, 200 mM NaCl, pH 8.0), 200 µl of 20% sodium dodecyl sulfate (SDS), 500 µl of phenol:chloroform:isoamyl alcohol (24:24:1), and 100 µl of 0.1 mm zirconia/silica. Samples were homogenized twice with a bead beater (BioSpec) for 2 min. After precipitation of DNA, crude DNA extracts were resuspended in TE Buffer with 100 µg/ml RNase and column purified to remove PCR inhibitors (BioBasic). Amplification of the V4 region (F515/R806) of the 16S rRNA gene was done^44^. Samples were sequenced on an Illuminia MiSeq platform (PE250). Sequence assembly, quality control, and clustering were performed using the usearch8.1 software package (http://www.drive5.com/usearch/). Reads were clustered into 97% ID operational taxonomic units (OTUs) using UPARSE algorithm^45^ followed by taxonomy assignment using the Silva database v128^46^ and the RDP Classifier^47^ with a bootstrap confidence cutoff of 80%. The OTU absolute abundance table and mapping file were used for statistical analyses and data visualization in the R statistical programming environment package phyloseq^48^.

### ELISA

CTX-I (IDS Immunodiagnostic Systems GmbH, Germany), OCN (Alfa Aesar, Germany) and undercarboxylated OCN (BlueGene, Japan) serum levels were measured by enzyme-linked immunosorbent assay (ELISA) according to the manufacturer’s instructions.

### Multiplex

IL-6, IL-10, IL-17, IFNγ, TGFβ, and TNFα serum levels were measured by Multiplex using a LEGENDplex Multi-Analyte Flow Assay Kit (BioLegend), according to the manufacturer’s instructions.

### Histology

Tibial bones were fixed in 4% formalin for 24 h and decalcified in EDTA (Sigma-Aldrich). Serial paraffin sections (2 μm) were stained for tartrate resistant acid phosphatase (TRAP) using a Leukocyte Acid Phosphatase Kit (Sigma) according to the manufacturer’s instructions. Osteoclast numbers were quantified using a microscope (Carl Zeiss) equipped with a digital camera and an image analysis system for performing histomorphometry (Osteomeasure; OsteoMetrics). For Calcein labeling, mice were injected with 30 mg/kg body weight of green fluorescent Calcein (Sigma) 11 and 4 days before sacrifice. Undecalcified bones were embedded in methacrylate and 5 mm sections were cut. Unstained sections were used to measure fluorescent Calcein-labeled bone surfaces at a wavelength of 495 nm. Toluidine Blue staining was performed for quantification of osteoblasts and von Kossa staining for bone mineralization.

### Micro-computed tomography

µCT imaging was performed using the cone-beam Desktop Micro Computer Tomograph “µCT 40” by SCANCO Medical AG, Bruettisellen, Switzerland. The settings were optimized for calcified tissue visualization at 55 kVp with a current of 145 µA and 200 ms integration time for 500 projections/180°. For the segmentation of 3D-volumes, an isotropic voxel size of 8.4 µm and an evaluation script with adjusted grayscale thresholds of the operating system “Open VMS” by SCANCO Medical was used. Volume of interest tibia: The analysis of the bone structure was performed in the proximal metaphysis of the tibia, starting 0.43 mm from an anatomic landmark in the growth plate and extending 1.720 mm (200 tomograms) distally.

### Collagen-induced arthritis

CIA was induced in 8-week-old female C57BL/6J or DBA/1J mice by subcutaneous injection at the base of the tail with 100 μl with 0.25 mg chicken type II collagen (Chondrex, Redmond, WA) in complete Freund adjuvant (Difco Laboratory, Detroit, MI), containing 5 mg/ml killed *Mycobacterium tuberculosis* (H37Ra). Mice were rechallenged after 21 days intradermal immunization in the base of the tail with this emulsion. The paws were evaluated for joint swelling and grip strength three times per week. Each paw was individually scored using a 4-point scale: 0, normal paw; 1, minimal swelling or redness; 2, redness and swelling involving the entire forepaw; 3, redness and swelling involving the entire limp; 4, joint deformity or ankylosis or both. In addition, grip strength of each paw was analyzed on a wire 3 mm in diameter, using a score from 0 to −4 (0, normal grip strength; −1, mildly reduced grip strength; −2, moderately reduced grip strength; −3, severely reduced grip strength; −4, no grip strength at all).

### K/BxN serum-induced model

K/BxN SIA was induced by intraperitoneal injection of 200 μl pooled K/BxN serum. The swelling of fore and hind paws were measured three times per week. Development of arthritis was evaluated for each paw using a semiquantitative scoring system (0–4 per paw; maximum score of 16).

### Postmenopausal bone loss model

At 6 weeks of age, mice were either sham-operated or ovariectomized. Surgically removed ovaries were examined histologically to verify successful ovariectomy. Mice were kept in identical housing conditions controlling for the same environment, food, light, and temperature for 8–10 weeks. Then tibial bones was excised and subjected to histological assessment or μCT imaging.

### Isolation and culture of osteoclast precursors

Bone marrow was isolated from 8-week-old C57BL/6J mice by flushing femoral bones with complete media. Bone marrow cells were plated in 96-well flat bottom plates (2 × 10^5^/well) in aMEM supplemented with 10% fetal calf serum, 30 ng/ml macrophages colony-stimulating factor, and 50 ng/ml RANKL (R&D Systems, Wiesbaden-Nordenstadt, Germany). Alternatively, osteoclast precursors were isolated from bone marrow-derived cell suspensions using CD11b microbeads (Miltenyi Biotec, Germany) according to the manufacturer’s instructions. The purity of isolated precursors was assessed by flow cytometric analysis using CD11b-FITC–labeled antibodies (Miltenyi Biotec). CD11b^+^ monocytes were plated in 96-well flat bottom plates (2 × 10^5^/well). Medium was changed after 72 h. Osteoclast differentiation was evaluated by staining fixed cells for TRAP using a Leukocyte Acid Phosphatase Kit (Sigma-Aldrich, Taufkirchen, Germany), according to the manufacturer’s instructions. Bone resorption assays were made using an Osteo Assay Surface 96 Well (Corning), according to the manufacturer’s instructions.

### SCFA measurements

Four to five replicates of frozen cecal samples (100 mg) or 50 µl of serum were weighed into a 2 ml polypropylene tube. The tubes were kept in a cool rack throughout the extraction. 33% HCl (50 µl for cecal contents or 5 µl for serum) was added and samples were vortexed for 1 min. One milliliter of diethyl ether was added, vortexed for 1 min, and centrifuged for 3 min at 4 °C. The organic phase was transferred into a 2 ml gas chromatography (GC) vial. For the calibration curve, 100 μl of SCFA calibration standards (Sigma) were dissolved in water to concentrations of 0, 0.5, 1, 5, and 10 mM and then subjected to the same extraction procedure as the samples. For GC mass spectrometric (GCMS) analysis, 1 μl of the sample (4–5 replicates) was injected with a split ratio of 20:1 on a Famewax, 30 m × 0.25 mm iD, 0.25 μm df capillary column (Restek, Bad Homburg). The GC-MS system consisted of GCMS QP2010Plus gas chromatograph/mass spectrometer coupled with an AOC20S autosampler and an AOC20i auto injector (Shimadzu, Kyoto, Japan). Injection temperature was 240 °C with the interface set at 230 °C and the ion source at 200 °C. Helium was used as carrier gas with constant flow rate of 1 ml/min. The column temperature program started with 40 °C and was ramped to 150 °C at a rate of 7 °C/min and then to 230 °C at a rate of 9 °C/min and finally held at 230 °C for 9 min. The total run time was 40 min. SCFA were identified based on the retention time of standard compounds and with the assistance of the NIST 08 mass spectral library. Full-scan mass spectra were recorded in the 25–150 *m*/*z* range (0.5 s/scan). Quantification was done by integration of the extracted ion chromatogram peaks for the following ion species: *m*/*z* 45 for acetate eluted at 7.8 min, *m*/*z* 74 for propionate eluted at 9.6 min, and *m*/*z* 60 for butyrate eluted at 11.5 min. GCMS solution software was used for data processing.

### Real-time PCR

Tissues were stored in RnaLater (Ambion) or directly transferred to TRIzol (Invitrogen). RNA was extracted according to the manufacturer’s instructions. Gene expression results are expressed as arbitrary units relative to expression of the house keeping gene glyceraldehyde 3-phosphate dehydrogenase (GAPDH). Primer sequences are as follows: TRAF6: 5′-AAAGCGAGAGATTCTTTCCCTG-3′ and 5′-ACTGGGGACAATTCACTAGAGC-3′; NFATc1: 5′-GGTGCCTTTTGCGAGCAGTATC-3′ and 5′-CGTATGGACCAGAATGTGACGG-3′; RANK: 5′-GCCCAGTCTCATCGTTCTGC-3′ and 5′-GCAAGCATCATTGACCCAATTC-3′; MMP9: 5′-GCTGACTACGATAAGGACGGCA-3′ and 5′-TAGTGGTGCAGGCAGAGTAGGA-3′; GPR41: 5′-ACCTGACCATTTCGGACCT-3′ and 5′-CCATCTCATGCCACATGC-3′; GPR43: 5′-GAGCAGCTGGATGTGGTACTG-3′ and 5′-CATGGGAACGAAAAACAGGA-3′; GPR109a: 5′-ATGGCGAGGCATATCTGTGTAGCA-3′ and 5′-TCCTGCCTGAGCAGAACAAGATGA-3′; GAPDH: 5′-GGGTGTGAACCACGAGAAAT-3′ and 5′-CCTTCCACAATGCCAAAGTT-3′.

### Western blotting

Cultured cells were washed twice with phosphate-buffered saline and subsequently lysed. Protein extracts were separated on 10% SDS-polyacrylamide gel, transferred on a nitrocellulose membrane, and stained with antibodies against TRAF6 (D-10, Santa Cruz, dilution 1:500), NFATc1 (7A6, Santa Cruz, dilution 1:500), pAMK (40H9, CellSignal, dilution 1:1000), and AMPK (34.2, abcam, dilution 1 :1000). An antibody against β-actin (AC-15, abcam, dilution 1 :25,000) was used as loading control. Uncropped scans of western blots are shown in supplementary information (Supplementary Fig. 5a).

### Extracellular flux assays

Bioenergetics of osteoclast precursors were determined using the XFe96 Extracellular Flux Analyzer (Seahorse Bioscience/Agilent Technologies, North Billerica, MA). Cells were seeded in specialized cell culture microplates at a density of 1 × 10^5^ cells/well and cultured for 24 and 48 h. One day prior to the measurement, a Seahorse XFe96 cartridge (Agilent/Seahorse Bioscience) was loaded with 200 µl XF Calibrant solution (Agilent/Seahorse Bioscience) per well and incubated overnight in a CO_2_-free atmosphere. One hour before the measurement, cells were incubated at 37 °C also in a CO_2_-free atmosphere. The ports of the Seahorse cartridge were loaded with glucose, oligomycin, and 2DG for the glycolysis stress test and oligomycin, FCCP, and antimycin A/rotenone for the mitochondrial stress test. After sensor calibration assays were run as detailed in the manufacturer’s manual recording extracellular acidification rate and oxygen consumption rate over time. Metabolic parameters were derived from the XF Wave software (Agilent/Seahorse Biosciences) and calculated with Microsoft Excel. All experiments were performed in pentaplicates. Raw values were normalized to the total protein content for each well.

### Flow cytometry

Spleens were smashed and filtered through a 40 μm gauze (BD Biosciences). Single-cell suspensions were stained for flow cytometry with the following antibodies and analyzed according to the gating strategy shown in (Supplementary Fig. 5b): CD4 Pacific blue (clone GK1.5, Biolegend, dilution 1:800), CD25 PE-cy7 (clone PC61, Biolegend, dilution 1:400), and FoxP3 AlexaFluor647 (clone MF-14, Biolegend, dilution 1:300) were used in combination with a FoxP3 staining kit (eBioscience).

### Effects of propionate on bone metabolism in humans

Healthy individuals (*n* = 7) received a daily oral supplementation of 1 mg/day proprionate (Propicum, Flexopharm Brain, Germany) for a period of 14 days. All analyses of human material were performed in full agreement with institutional guidelines and with the approval of the Ethical committee of the University Hospital Erlangen (permit # 148_16B). Informed consent and permission to use the obtained data for research were obtained from all subjects. Serum was taken at baseline and after 14 days and analyzed for C-terminal collagen type I cleavage products (CTX-1) as marker for bone resorption and OCN as marker for bone formation by ELISA.

### Statistical analysis

Data are expressed as mean ± s.d unless otherwise indicated in the figure legends. All relevant data are available from the authors. Analysis was performed using Student’s *t*-test, single comparison, or analysis of variance (ANOVA) test for multiple comparisons (one-way or two-way ANOVA followed by Tukey’s or Bonferroni’s multiple comparisons test, respectively). All experiments were conducted at least two times. *P*-values of 0.05 were considered significant and are shown as *p*<0.05 (*), *p*<0.01 (**), or *p*<0.001 (***). Graph generation and statistical analyses were performed using the Prism version 4c software (GraphPad, La Jolla, CA).

### Data availability

All relevant data are available from the authors upon reasonable request. 16S RNA sequencing data are deposited in publicly accessible database and available under the following accession code: PRJNA419741.

## Electronic supplementary material

Supplementary Information
