# Supplementary figures and images for: Short-chain fatty acids regulate systemic bone mass and protect from pathological bone loss
Source: Nat Commun. 2018 Jan 4;9:55. doi: 10.1038/s41467-017-02490-4 (PMC5754356; doi:10.1038/s41467-017-02490-4)

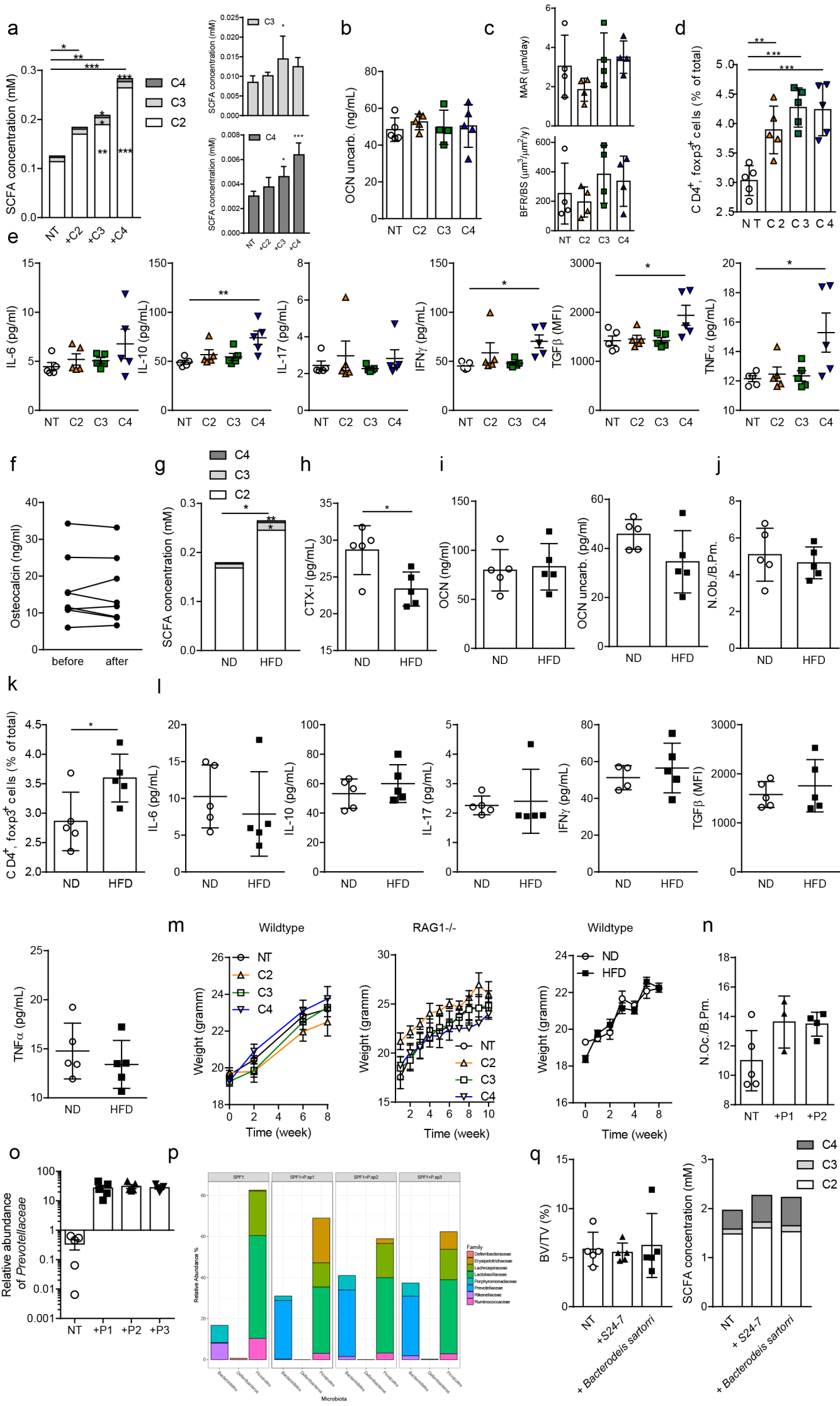


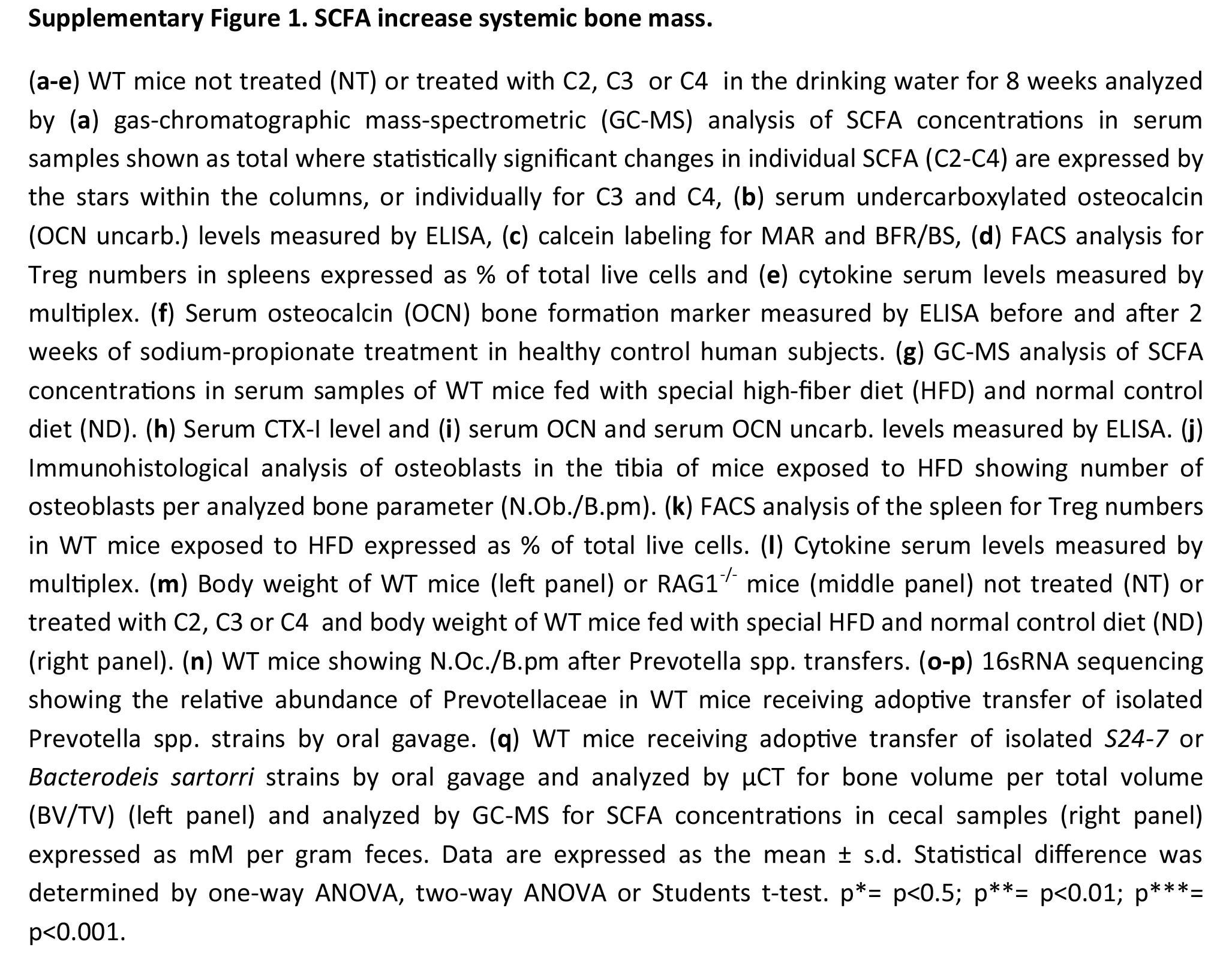


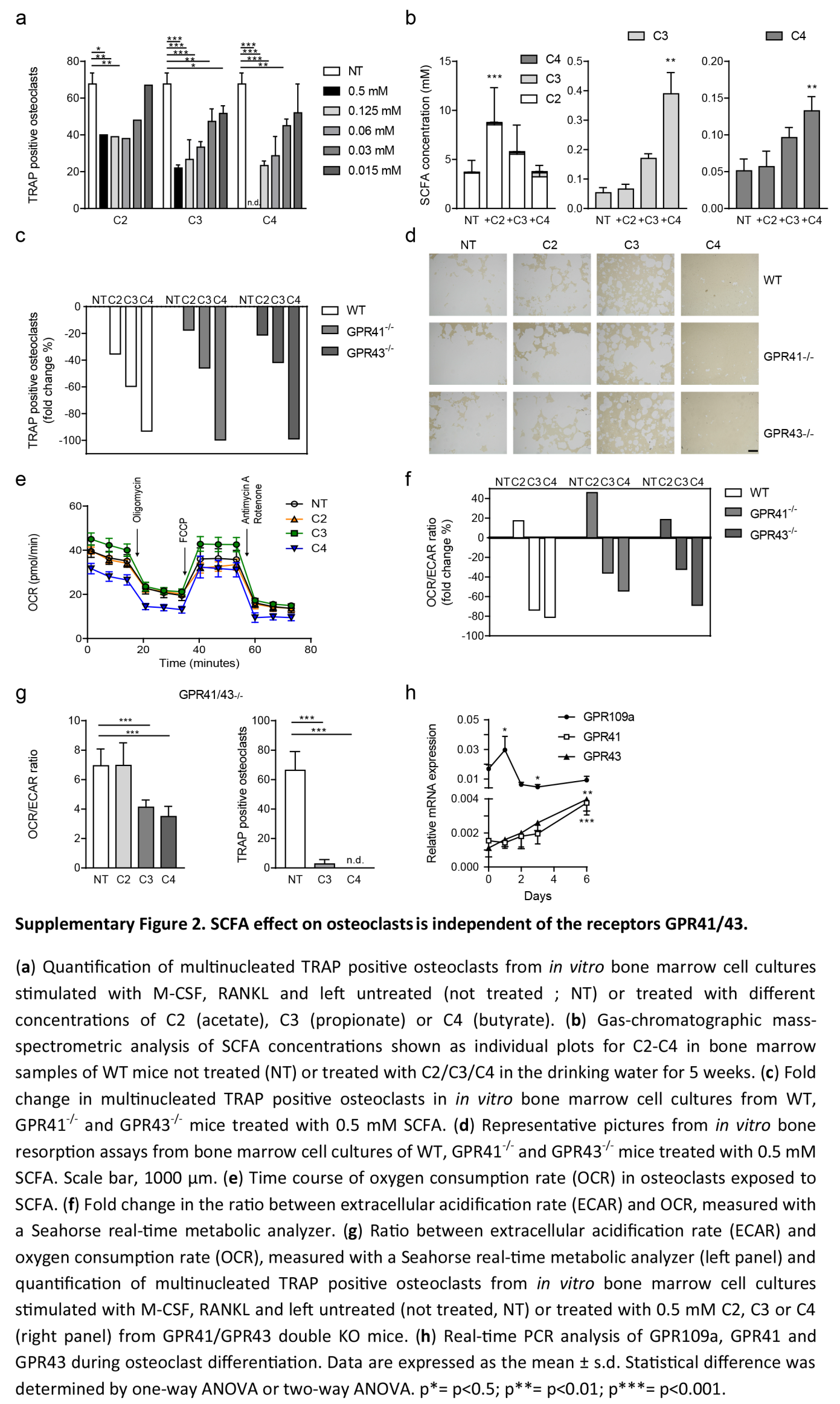


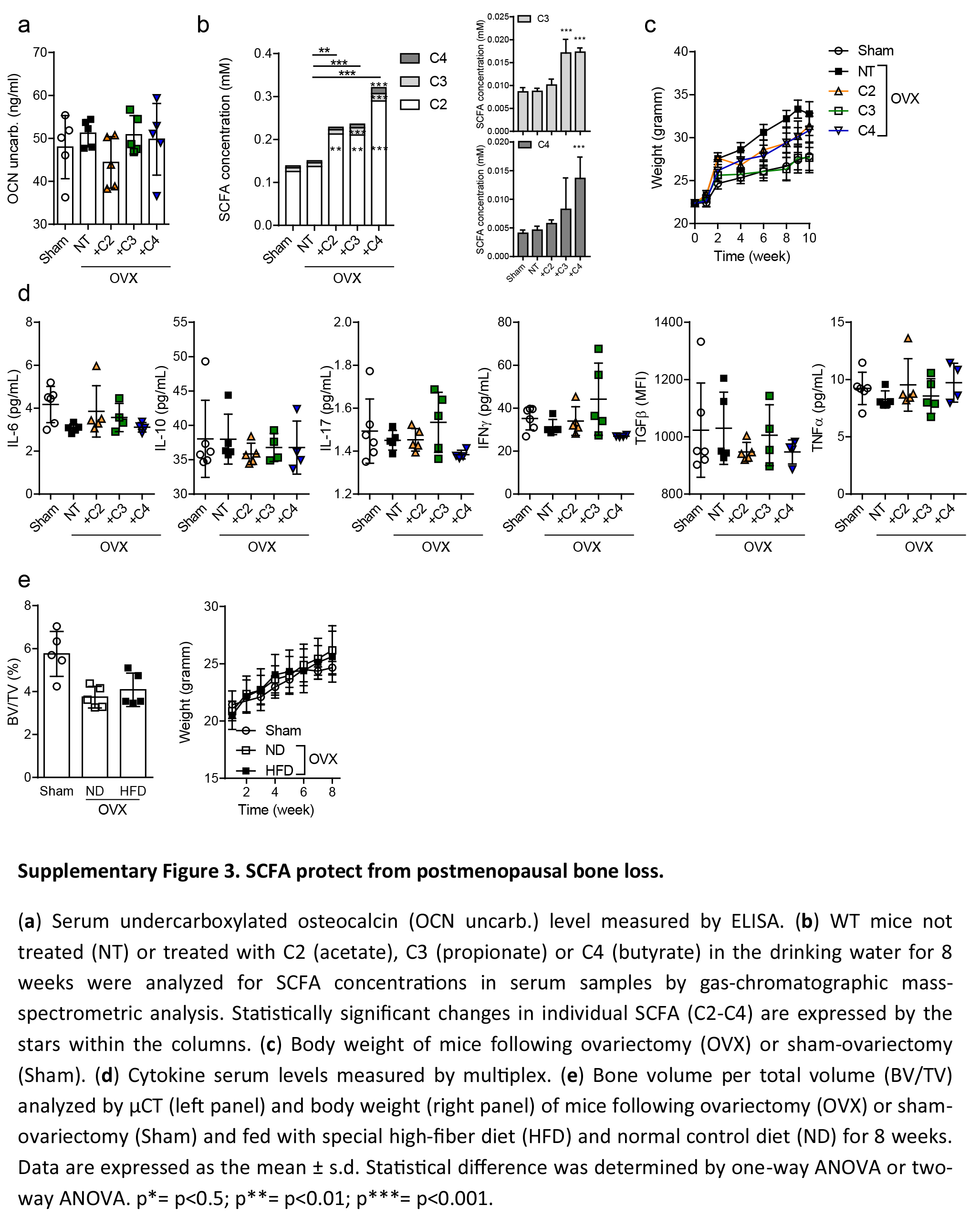


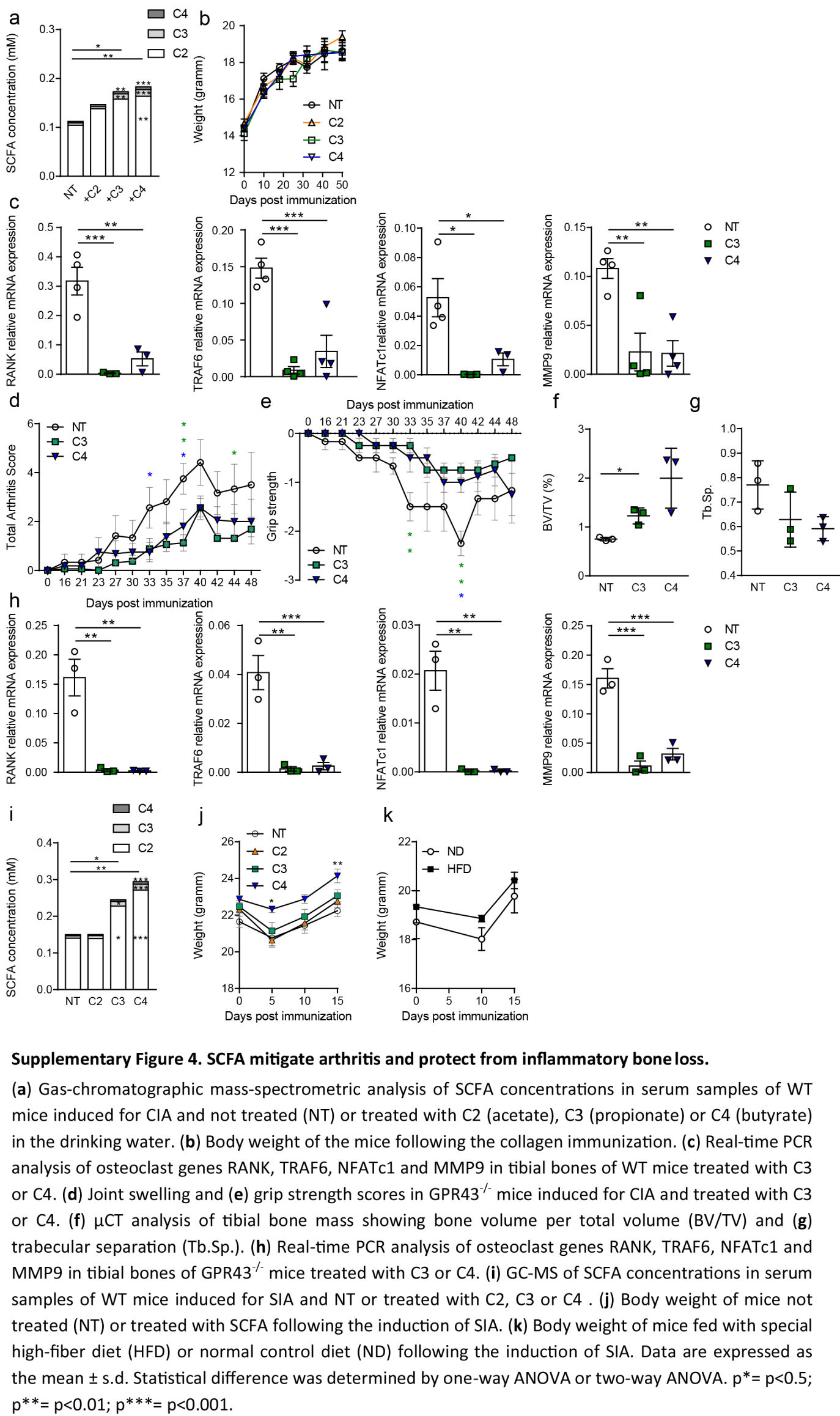


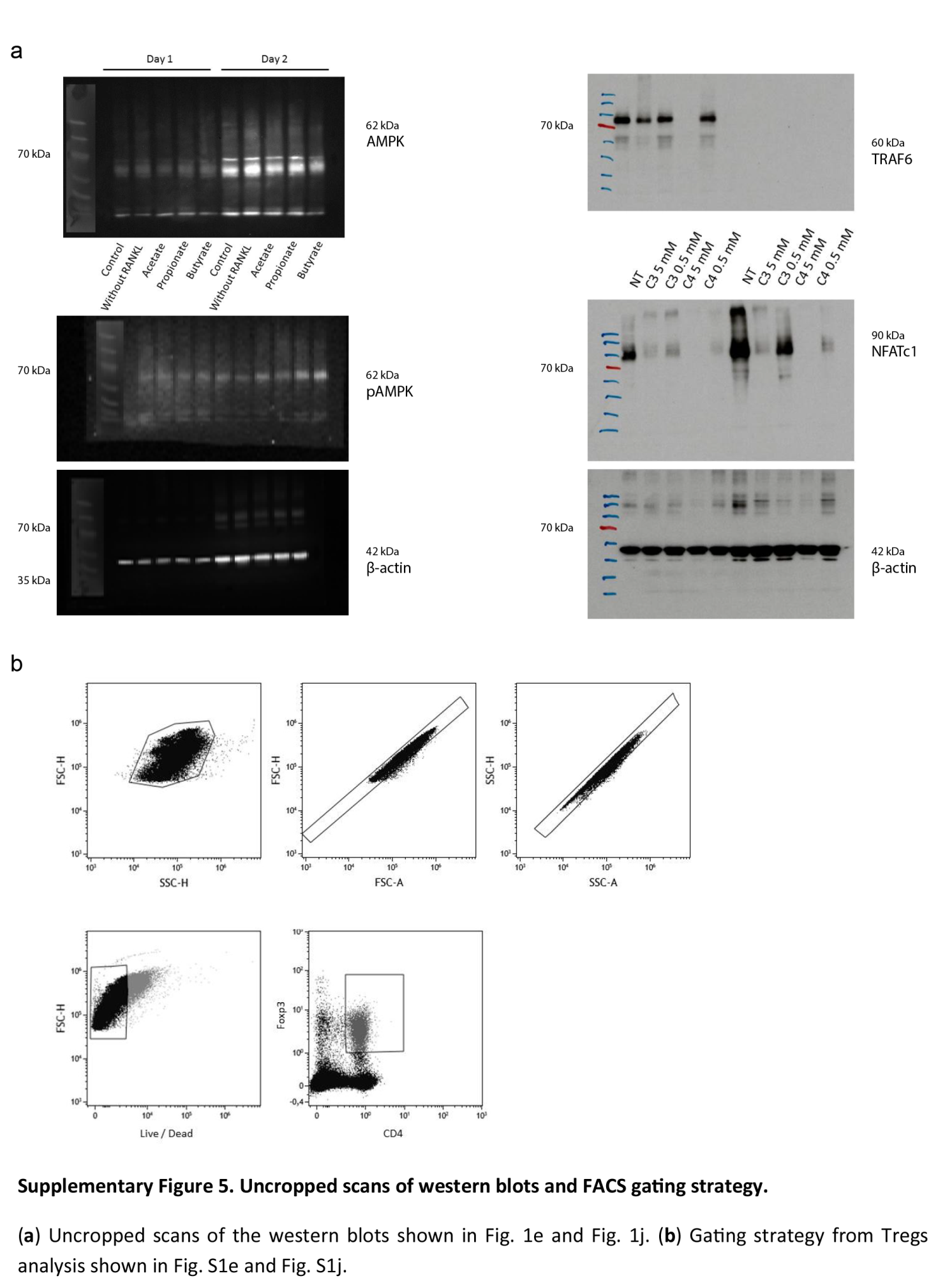


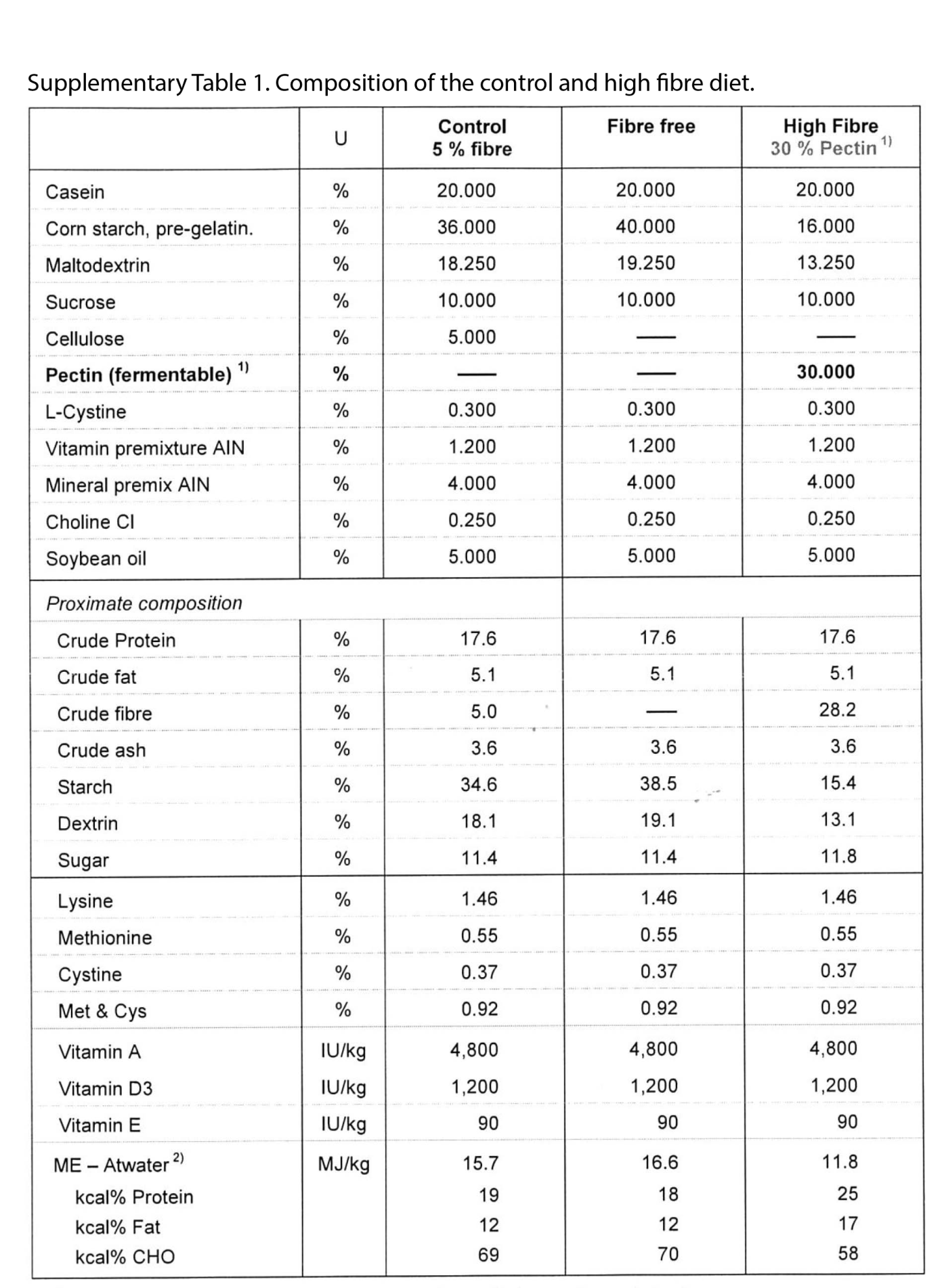

Supplement: Supplementary file 1 — Supplementary Information [file 41467_2017_2490_MOESM1_ESM.docx]
